# Personalized Care for Patients with Cancer in the Precision-Medicine Era

**DOI:** 10.3390/ijerph20043023

**Published:** 2023-02-09

**Authors:** Luís Carlos Lopes-Júnior, Luciana Chain Veronez

**Affiliations:** 1 Health Sciences Center, Federal University of Espírito Santo (UFES), Vitória 29043-900, ES, Brazil; 2 Ribeirão Preto Medical School, University of São Paulo (USP), Ribeirão Preto 14049-900, SP, Brazil

Important advances in cancer management have been made in the beginning of the 21st century. Most of them are associated with patient care and treatment, including development of new or more precise individual therapies and molecular diagnostics, which had implicated in better outcomes and extended survivals, mostly due to personalized approaches for each tumor and patient into the precision-medicine (PM) era [1,2].

In 2011, the term PM was introduced as the “tailoring of medical treatment to the individual characteristics of each patient” by the National Academies of Sciences, Engineering, and Medicine, which also proposed the use of omics data to improve patient care, including genomics, pharmacogenomics, transcriptomics, proteomics, epigenomics, metabolomics, and microbiomics [3]. In other words, PM refers to the medical management personalized and based on every single patient in order to achieve the best health-care, including the most effective treatments and diagnostics. This also implies in a standard and more precise patient-care, mainly related to the advances in data collection, analytics, and information [4].

PM-related technologies contribute to make the Research & Development (R&D) process more efficient, either by reducing product development time or by increasing its success rate. In order to be effective and successful, this approach assumes the integration of several areas of interdisciplinary knowledge and advanced technologies focused on patient’s characteristics and specific healthy needs, including omics sciences, bioinformatics, biomarkers, biomedical approaches, digital health, data sharing, and data science [5]. In this context, in recent years, PM has begun a new era in medicine focused on the *P4 Medicine: Participatory, Preventive, Predictive, and Personalized methods*, which is shifting patient care [5].

In oncology, PM is emerging as the tumor treatment and prevention that consider the cancer variability in terms of gene expression patterns, tumor specific (immune) microenvironment, and patients’ particular lifestyles and morbidities [6]. Since precision medicine in oncology (PMO) applies genomic and other molecular analyses of tumor biopsies, it has the potential to optimize cancer therapy, taking into account the tumor response and drug resistance for each patient [6,7], ensuring a better quality of life and a better patient care [6].

As PMO introduces a big data analysis of personalized omics approaches, there are still challenges in the translation of all these data into meaningful and equitable benefits to patients and health care. Issues regarding adequate clinical trial design, elevated costs, data analysis, and equity also remain to be solved [8] in order not only to deliver more effective therapeutics, but also a personalized patient-centered care [9,10,11].

The recognition that each patient with cancer is unique is not a new concept for heathy professionals. Each patient presents its specific preferences, needs, tolerances, and unique tumor vulnerabilities even when suffering from very similar diseases or course of treatment, which demands and highlight the importance of the PM approach and the personalized care [12,13].

In this regard, it should be highlighted that the successful implementation of PMO requires interprofessional collaboration, which also includes nurses [11]. These professionals are the ones who are strongly prepared to not only conduct clinical and translational scientific research, but also engage with patients and families, as well as integrate and incorporate the omics sciences and bioinformatics into clinical practice in order to help in delivering the PMO [11,14,15].

This Special Issue of the *International Journal of Environmental Research and Public Health* invited manuscripts that address topics relating to personalized cancer care into the precision-medicine era. We welcome manuscripts: (i) reporting the evaluation of experiments, observational studies as well as randomized trials; (ii) investigating physiology, integrating omics sciences, bioinformatics and biomarkers as well as mechanisms linking environment and health outcomes for cancer patients. Original articles, experimental studies, systematic reviews and metanalyses, scoping reviews, methodological studies are also welcome. The findings should have implications for the improvement of personalized nursing and clinical care, PMO, nursing education, personalized cancer care, and nursing management, as well as contributing to the construction of education, research and health policies.

We hope to contribute to the disclosure of the importance of the personalized cancer care and to provide an interesting and innovative reading experience with this Special Issue. We thank in advance all the authors, the editorial office professionals, the journal and publication platform for providing us with the opportunity to collect multi-disciplinary work covering this important topic in the precision-medicine era.

Hence, it is time to make cancer care beyond technically sophisticated, especially personalized, holistic and patient-centered care. Indeed, precision medicine in oncology can offer a chance to provide better patient outcomes with improvement in the quality of life, as well as in the quality of cancer care. Additionally, the promise of PMO can be achieved within the context of patients’ preferences, supporting by the health care system, leading to better health outcomes as well as better health system performance.

## Data Availability

Not applicable.
